# Swirlonic state of active matter

**DOI:** 10.1038/s41598-020-73824-4

**Published:** 2020-10-08

**Authors:** Nikolai V. Brilliantov, Hajar Abutuqayqah, Ivan Yu Tyukin, Sergey A. Matveev

**Affiliations:** 1 Skolkovo Institute of Science and Technology, Moscow, Russia; 2 Department of Mathematics, University of Leicester, Leicester, LE1 7RH UK; 3 Marchuk Institute of Numerical Mathematics, RAS, Gubkin st. 8, Moscow, Russia; 4 Saint Petersburg Electrotechnical University “LETI”, Professora Popova 5, St. Petersburg, Russia

**Keywords:** Applied mathematics, Computational science, Statistical physics, thermodynamics and nonlinear dynamics

## Abstract

We report a novel state of active matter—a swirlonic state. It is comprised of *swirlons*, formed by groups of active particles orbiting their common center of mass. These quasi-particles demonstrate a surprising behavior: In response to an external load they move with a constant velocity proportional to the applied force, just as objects in viscous media. The swirlons attract each other and coalesce forming a larger, joint swirlon. The coalescence is extremely slow, decelerating process, resulting in a rarified state of immobile quasi-particles. In addition to the swirlonic state, we observe gaseous, liquid and solid states, depending on the inter-particle and self-driving forces. Interestingly, in contrast to molecular systems, liquid and gaseous states of active matter do not coexist. We explain this unusual phenomenon by the lack of fast particles in active matter. We perform extensive numerical simulations and theoretical analysis. The predictions of the theory agree qualitatively and quantitatively with the simulation results.

## Introduction

Active matter is a substance comprised of active particle that demonstrate the motility, that is “the ability to exhibit motion and to perform mechanical work at the expense of metabolic energy”^1^. The active particles consume energy from the environment and drive themselves far from equilibrium^2,3^. Living matter (biological systems) provides an uncountable amount of examples of active particles systems^4^. Other examples refer to robotics^5^, biomedicine^6^, and social science^7,8^; an interesting laboratory realization of artificial active particles are the so-called vibrots^9–13^. Similarly to common molecular systems active matter undergo different phase transitions – separation into dense and dilute phase^9,10^ and crystallization^14–16^; these phenomena my be described within the framework of conventional thermodynamics^14–18^.

The most prominent feature of systems of active (self-propelled) particle is the formation of self-organized coherent structures, see e.g.^2,3,19–29^. Among these are intriguing milling patterns emerging in circular motion, when a group of individuals follow one another around an empty core. Such swirling patterns have been observed for animals at different evolution stages, ranging from plant-animal worms and insects to fish^30–40^. To describe the swirling motion several models have been proposed, including the celebrated Vicsek^41–43^ and Vicsek-like models^44–47^, as well as models based on the inter-particle interaction potential^20,29,48–52^. Although the inter-particle potential between active particles (agents) does not exists in reality, it mimics an *intention* of an agent to change its velocity according to some rules. It is convenient to put these rules in the form of Newtonian forces acting between the agents. Alternatively, the rules may be explicitly written in terms of velocity of an agent at the next time step, that is, determined by the velocities and coordinates of the (usually the nearest) neighbors. Below we will use the former approach, based on the inter-particle potential. Note that here we address the swirling motion of active particles; swirling also exists in a system of agitated passive particles, which have been studied in^53^.

The milling motion reported in Refs.^20,29,48–53^ referred to a single pattern where particles orbited around a common center. The number of particles was not large—it did not exceed a few hundreds. Formation of multiple milling patterns has not been reported. A possible reason for this could be a small system size due to the computational constrains. Indeed, the system of dynamic equations for active particles forms a set of very stiff ordinary differential equations (ODEs). An accurate convergent solution requires in this case an extremely small time step^54^. Hence it seems interesting to simulate much larger systems of active particles to explore the formation of multiple structures and their evolution.

In the present study we investigate large systems of active particles and report the formation of the conventional phases—gaseous and liquid phase, as well as solid phase, but also a new state of active matter—“swirlonic” state. In a gaseous state the active matter tends to occupy all the available space, while in a liquid state its volume is limited. However, in contrast to common molecular systems, we did not observe the gas–liquid coexistence, but only the presence of a single phase. In the swirlonic state multiple milling patterns—swirlons are formed. The swirlons behave like individual super-particles with surprising properties. They attract each other and coalesce upon collision, forming swirlons of a larger mass. When an external force is applied to a swirlon, it moves in the direction of the force, with a *constant velocity*, proportional to the applied force, as a particle in viscous medium. The steady velocity of the swirlon is inversely proportional to the mean-square velocity of the orbital motion of particles comprising the swirlon. The larger the swirlon, the larger the mean-square velocity and hence smaller the steady velocity. In other words, the mobility of swirlons decays with its mass. The swirlonic state is not a stable but transient state, since eventually all swirlons should collapse into a single milling structure. However the mobility of heavy swirlons drastically decreases, which entails, for a large system, a diverging transient time from the swirlonic state to the single milling state. This makes the swirlonic state of a special interest.

To study large systems of active particles we slightly modify the repulsion part of the commonly used Morse interaction potential. Namely, we apply the Gaussian dependence of the potential on the inter-particle distance, instead of previously used exponential dependence. This simple modification leads to a drastic reduction of the stiffness of the ODEs describing the system and keeps, at the same time, qualitatively the same behavior. The reduced ODE stiffness allowed to release very severe restrictions for the size of the computation time step and simulate relatively large systems, up to few tens of thousand particles. Simulation of such large system gives a new insight into the properties of active matter.

## Model

We consider a two-dimensional system comprising *N* identical self-driven particles of mass $m=1$. Then the equation of motion of *i*th particle reads,

1 $$\begin{aligned} \dot{{\varvec{r}}}_i &= {\varvec{v}}_i \end{aligned}$$

2 $$\begin{aligned} m \dot{{\varvec{v}}}_i & = {\varvec{f}}_i +(b-v_i^2) {\varvec{v}}_i +{\varvec{g}}_i, + 2\sqrt{D}{\varvec{\xi }}_i(t) \end{aligned}$$

where ${\varvec{r}}_i$ and ${\varvec{v}}_i$ are the coordinate and velocity of the *i*-th particle, ${\varvec{f}}_i$ is a force acting from all other particles, ${\varvec{g}}_i$ is an external force and ${\varvec{\xi }}_i(t)$ is a random force, modeled by a Gaussian white noise,

3 $$\begin{aligned} \left\langle {\varvec{\xi }}_i(t) \right\rangle =0,\qquad \left\langle \xi _i^{\alpha }(t) \xi _j^{\beta } (t^{\prime }) \right\rangle = D \delta _{ij} \delta _{\alpha \beta } \delta (t-t^{\prime }), \end{aligned}$$

where $\alpha$ and $\beta$ denote the Cartesian components of the vector ${\varvec{\xi }}_i$

The self-driving force $(b-v^2) {\varvec{v}}$ describes the motion of the active particle with a constant velocity $v= \sqrt{b}$ in the absence of all other interactions. We use the following model for the interaction with other particles:

4 $$\begin{aligned} {\varvec{f}}_i & = -{\varvec{\nabla }}_i \sum _{i\ne j}^N U\left( |{\varvec{r}}_i - {\varvec{r}}_j | \right) \end{aligned}$$

5 $$\begin{aligned} U & = U_r(r) + U_a(r) = C_r e^{-(r/l_r)^2} - C_a e^{-r/l_a} . \end{aligned}$$

$U_r(r)$ and $U_a(r)$ refer respectively to the repulsive and attractive part of the interaction potential with the according constant $C_r$ and $C_a$ characterizing the interaction strength. $l_r$ and $l_a$ describe the characteristic lengths of $U_r(r)$ and $U_a(r)$. As we have already mentioned, we use the Gaussian dependence on distance for the repulsive part of the potential, instead of exponential dependence of the Morse potential. Eq. (2) has the form of stochastic Langevin equation. The corresponding Fokker–Planck equation for the distribution function $\Phi ( {\varvec{r}}, {\varvec{v}})$ is given in the Appendix A; it can be derived using the standard techniques, see e.g.^55, 56^.

The dynamic of the system is described by *N* Eqs. (1)–(4) for $i=1,\ldots N$ along with Eqs. (4)–(5) for the interaction potential. We simulate two-dimensional systems. In simulations we use up to 38,000 ODE, different sets of parameters $(C_a,l_a,C_r,l_r)$ and different initial densities of particles and system size. We start with random configurations and random velocities, randomized both in magnitude and direction. Depending on the above parameters one obtains rather different behavior of the system, which is qualitatively the same as reported in Refs.^29,52^, that is, flocking, swarming and milling. However, additionally, a novel state with many milling structures has been detected, which we call “swirlonic state”. We have checked that this state emerges also for other potentials, with attractive and repulsive parts, including Morse potential. Still an accurate modeling of such states with multiple swirlons is computationally very challenging. Here we report our findings for the soft (Gaussian) form of the repulsive potential, see Eq. (5). The results have been obtained for a system in liquid, gaseous and swirlonic state confined in a circular region.

## Phases of active matter

**Figure 1:**
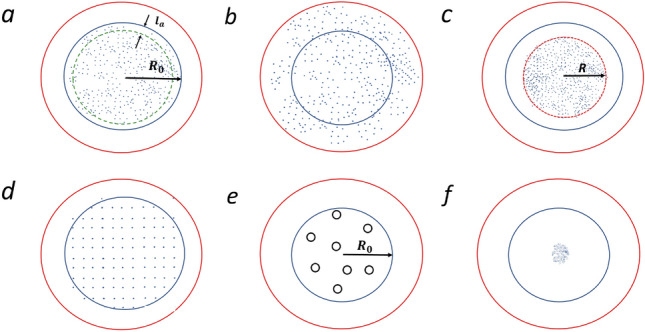
For varying density and parameters of interaction potential different states of active matter are obtained: (**a**) Initial state of a uniform density and random velocities, the system is confined in a circle of radius ${R_0}$; (**b**) “gaseous” phase, where the particles occupy all the available space; (**c**) liquid state, where only a part of available space is occupied (note the lack of the liquid–gas coexistence); (**d**) solid phase, where the mobility of particles is suppressed; (**e**) swirlonic phase, comprised of swirlons—“super-particles” built up by orbiting around a common center active particles; (**f**) collapsed state, where a single very dense structure is formed.

Starting from a uniformly distributed particles, confined in some area, Fig. 1a different phases may be formed depending on the density and parameters of interaction potential. If the density is low, so that the average distance between the particles exceeds the characteristic length of the interaction potential, the interactions are weak and compact patterns do not emerge. The particles run away from their initial position and occupy all the available space. They move chaotically, and thus form a “gaseous” phase, Fig. 1b. In contrast, for larger density, when many particles are located within the interaction distance, the attraction is stronger, and a state with a fixed density emerges, Fig. 1c. In this case the active matter does not occupy all the available volume, but only part of it. This is a “liquid” state. Interestingly, we did not observe coexistence of two phases—gaseous and liquid as in molecular systems. This may be explained by the fact that in contrast to molecular systems, where the distribution function for particles’ velocities is Maxwellian, the according distribution for active particles decays much faster for large velocities. Namely it reads (see the Appendix A):

6 $$\begin{aligned} \Phi (v) \sim e^{-v^4/2D} \qquad {\mathrm{for}} \qquad v \rightarrow \infty \end{aligned}$$

Hence, while the fast particles are abundant in molecular systems, they are practically lacking in active matter. These fast particle can overcome the attraction forces of the molecular surrounding and give rise to the gaseous phase; the lack of fast particles in active matter results in the lack of the gas, coexisting with the liquid phase. If the particle density is still larger and the repulsive forces are strong, the active matter falls into a solid phase, Fig. 1d, where the particles mobility is suppressed.

In some range of parameters a novel swirlonic phase is formed, Fig. 1e. It is comprised of swirlons—“super-particles” with many astonishing properties which we address below. Individual active particles in swirlons perform a swirling motion around their common center. As we demonstrate in what follows, the swirlons are formed when local fluctuating force exceeds the critical force, which characterizes the ability of an active particle to move against an applied load.

Finally, for large density and very strong attractive forces a collapsed state is observed, Fig. 1f. In the collapsed state active particle also move around the common center, however the character of the motion is rather irregular. In the present study we will focus on the gaseous, liquid and especially on the swirlonic phase.

## Qualitative theory of phase behavior

### Critical force

To understand the conditions for the formation of different pases we consider first a single active particle under an action of a constant force. It obeys the equation (recall that $m=1$):

7 $$\begin{aligned} \dot{ {\varvec{v}}} = b {\varvec{v}} - v^2 {\varvec{v}} + {\varvec{f}} \end{aligned}$$

**Figure 2:**
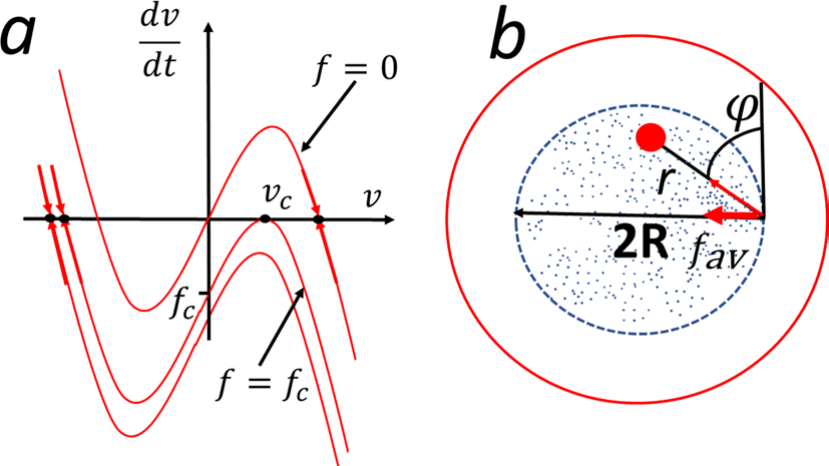
(**a**) The dependence of *dv*/*dt* on *v* for different force strengths (from top to bottom): $f=0$, $f=f_c$ and $|f|>|f_c|$. The stable points towards the system evolves are shown. For $|f|\le |f_c|$ there are two stable points $dv/dt=0$ – one with the velocity in the direction of the force and another in the opposite direction. For $|f|\ge |f_c|$ the only solution corresponds to the velocity in the direction of the force. (**b**) Illustration of the derivation of the average force $f_{\rm av}$ that keeps the particles within the initially occupied area (the circle of radius *R*). Due to symmetry the average force is directed towards the center of the circle.

For simplicity we assume that the direction of the force and velocity coincide, then Eq. (7) is a scalar equation. In Fig. 2a the dependence $dv/dt=0$ on *v* is demonstrated for different *f*. It shows that the system evolves to states with $dv/dt=0$, which are depicted as stable points in the figure. These are the roots of the equation, $bv-v^3+f=0$. For $|f|\le |f_c|$ there are roots, which correspond to the steady motion in the direction of the force and in the opposite direction. For $|f|\ge |f_c|$ there is only one root, corresponding to the steady motion in the direction of the force. The critical force reads,

8 $$\begin{aligned} f_c = v_c^3-bv_c =- \frac{2b^{3/2}}{3 \sqrt{3}} , \end{aligned}$$

where $v_c=\sqrt{b/3}$ is the critical velocity, which is the root of the equation, $d(bv -v^3+f)/dv=0$, see Fig. 2a. One can interpret this results as follows: If the applied force *f* does not exceed the critical force $f_c$ an active particle can move against the force, that is, such force would not prevent the escape of the particle from some area, where such force is acting. In contrast, if *f* is larger than the critical force, an active particle can not escape from the region where this force is acting. The above estimate of the critical force will be used to estimate the conditions for different phase formation.

### Conditions for “gaseous” phase

Now we estimate the average force acting on active particles at different locations. Let *N* active particles are *uniformly* distributed within a circle of radius *R*, see Fig. 1c (note that *R* may differ from the initial value $R_0$). If $l_a, \, l_r \ll R$ the average force, acting on a particle from its neighbors, in the internal part of the circle is zero. At the same time particles near the border experience uncompensated average force $f_{\mathrm{av}}$ directed towards the circle center, see Fig. 2b. Consider a particle located exactly at the border, then the average force may be estimated as

9 $$\begin{aligned} f_{\mathrm{av}} =2 \int _0^{\pi /2} d\phi \int _0^{2R \sin \phi } r dr n\, f(r) \sin \phi . \end{aligned}$$

Here $n=N/(\pi R^2)$ is the number density of particles, $n rdr d\phi$ gives the amount of particles within a small surface element $rdrd\phi$ which act on the chosen particle at the border with the force $f(r) = (C_a/l_a) e^{-r/l_a} - 2 (rC_r/l_r^2) e^{-r^2/l_r^2}$. The factor $\sin \phi$ accounts for the fact that only the component of the above force, directed towards the circle center, contributes to the average force; the component normal to this direction does not contribute due to the symmetry, see Fig. 2b. As it follows from Eq. (9) both the repulsive and attractive forces contribute to $f_{{\rm av}}$, but these act in opposite directions. Since we focus in our study on the swirlonic states, which require that $l_a > l_r$ (see also the classification of Ref.^52^) we can neglect the repulsive force to find an upper estimate for $f_{av}$. Then the integration over *r* in Eq. (9) yields,

10 $$\begin{aligned} f_{\mathrm{av}}& = 2 C_an l_a \int _0^{\pi /2} \sin \phi d\phi \left[ 1- \left( 1+\frac{2R\sin \phi }{l_a} \right) e^{-2R\sin \phi /l_a} \right] \nonumber \\ & \simeq 2 C_a n l_a \left[ 1- \int _0^{\infty } \phi \left( 1+ \frac{2R\phi }{l_a} \right) e^{-2R\phi /l_a} d\phi \right] \nonumber \\ & = 2 C_a n l_a \left[ 1- \frac{3}{4} \frac{l_a^2}{R^2} \right] . \end{aligned}$$

Writing the second line in the Eq. (10) we assume that $R/l_a \gg 1$, which implies that only $\phi \simeq \sin \phi \ll 1$ contribute from the part of the integrand with the exponential factor; this also allows to change the upper limit $\pi /2$ to $\infty$. Although the above estimate is justified for $R/l_a \gg 1$, it works, in practice, rather well (with the accuracy better than 17%) already for $R \ge l_a$. We assume that a particle remains in a condensed phase if the average force is larger than the critical force. Then from Eqs. (10) and (8) follows the estimate for the condition of the condensed phase:

11 $$\begin{aligned} C_a n l_a \left[ 1- \frac{3}{4} \frac{l_a^2}{R^2} \right] > \alpha b^{2/3} , \end{aligned}$$

where $\alpha \sim \mathcal{O}(1)$ is the constant of the order of one. If the condition (11) is not fulfilled, the “evaporation” takes place and the system evolves to the “gaseous” state, occupying all available space, see Fig. 1b. The evaporation is facilitated for small systems, at low density, with a weak, short-range potential. The evaporation through the flat boundary, $R\rightarrow \infty$, occurs at $b^{2/3} \sim \alpha C_a n l_a$. With Eq. (8) for the critical force, the condition for condensed state may be written as

12 $$\begin{aligned} \phi _a > \alpha f_c^{*}, \end{aligned}$$

where $f_c^{*} = f_c l_a/C_a$ is the reduced critical force and $\phi _a =\pi n l_a^2$ is the effective “packing fraction”, associated with the length of attractive interactions $l_a$ and particle number density $n=N/\Omega$, where $\Omega =\pi R^2$ is the area occupied by the active matter. The latter can differ from the initial area as well as from the total available area, see Fig. 1a and c.

**Figure 3:**
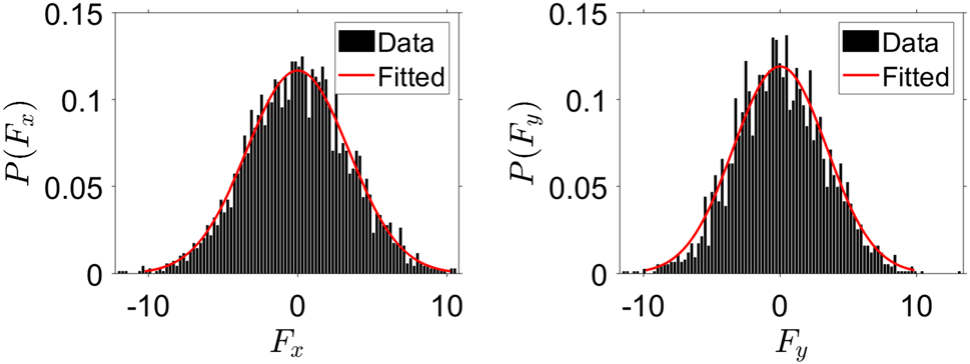
An example of the local force distribution *P*(*F*) at the initial stage of the system. The active particles interact with the inter-particle potential (5). Lines-gaussian fit $P(F_i)=Ae^{- F_i^2/(4an)}$, as it follows from (13), for the *x*-component ($i=x$, left panel) and *y*-component ($i=y$, right panel). $A=0.116$ is a fitting parameter. Data are the average over 50 runs. The system parameters are $C_a=1$, $l_a=1.3$, $b=0.8$, $D=0$, $C_r=0$, $R_0=8$ and $N=3000$

### Conditions for liquid and swirlonic state

Now we need to discriminate between two condensed states–liquid, with a uniform density, occupying a fixed volume, Fig. 1c, and swirlonic, with multiple milling structures, Fig. 1e. That is, we need to find the condition, when a uniform state becomes unstable. We notice that swirlons emerge due to a “local capture” of active particles by their surrounding. Indeed, these structures emerge, when particles remain localized in the vicinity of their initial positions. In other words, it is expected that there exist local forces with the strength exceeding the critical force $f_c$. Since the average force is zero for uniformly distributed particles, the arising forces have fluctuating nature. Let us estimate these fluctuating forces.

Consider a particle in the internal part of the system. The number of neighbors that act on the particle is about $\sim n l_a^2$ and they act on the particle with the force of the order $\sim (C_a/l_a)$ (again we neglect the repulsive forces). The average force is zero, but due to the density fluctuations, which may be estimated as $\sim \sqrt{n l_a^2}$ a non-compensated fluctuating force of the order of $\sim (C_a /l_a) \sqrt{n l_a^2} \sim C_a n^{1/2}$ will arise.

A more rigorous analysis presented in Appendix B confirms the above estimates. Namely, it may be shown that the distribution of the local fluctuating force ${\varvec{F}}$ in a space uniform system with the interaction potential $U= C_ae^{-r/l_a}$ reads as shown in Fig. 3 (see the “Appendix B”),

13 $$\begin{aligned} P({\varvec{F}}) \simeq \frac{1}{4 \pi an} e^{-{\varvec{F}}^2/(4an)}; \qquad a\equiv \frac{\pi }{8} C_a^2 . \end{aligned}$$

The average force may be obtained from the above distribution (13) as

14 $$\begin{aligned} \left\langle |{\varvec{F}} |\right\rangle & = \int F P({\varvec{F}}) d {\varvec{F}} = 2\pi \int _0^{\infty } F\cdot F \frac{1}{2 an}e^{- F^2/(4an)} dF = \frac{\pi }{2\sqrt{2}} C_a n^{1/2} . \end{aligned}$$

This average force is to be compared with the critical force $f_c$, (8), to escape from the local surroundings. Hence we can write the estimate for the condition that an active particle is locally caught by the fluctuating force and participate at the formation of a swirlon:

15 $$\begin{aligned} C_a n^{1/2} > \alpha _1 b^{2/3}, \end{aligned}$$

where $\alpha _1=\mathcal{O}(1)$ is the constant of the order of one. The above condition (15) does not, however, discriminate between swirlonic and collapsed state, but rather demarcates liquid and swirlonic states. With the above notations the condition of liquid phase may be put into the form,

16 $$\begin{aligned} \phi _a> \alpha f_c^{*} \qquad \& \qquad \phi _a^{1/2} > \alpha _1 f_c^{*} . \end{aligned}$$

**Figure 4:**
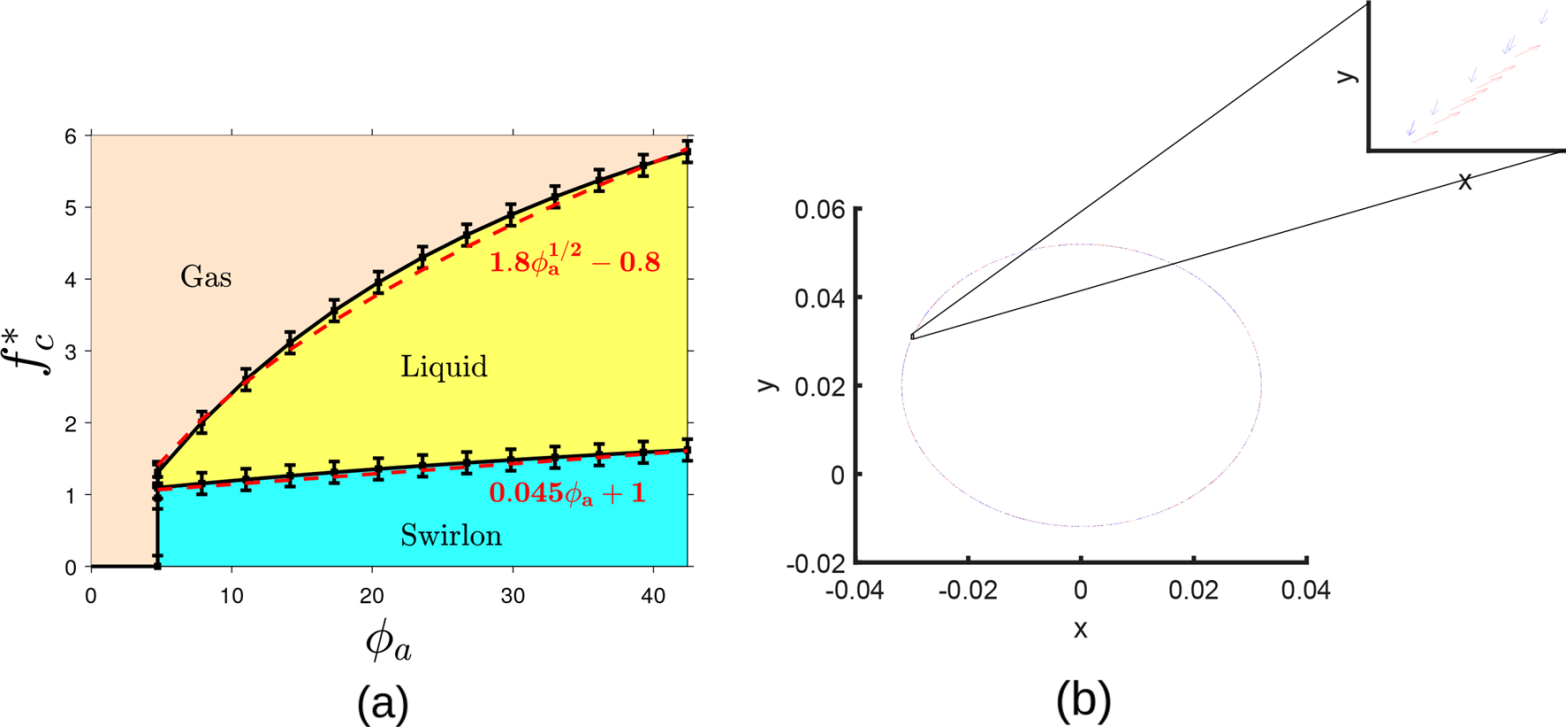
(**a**) Phase diagram of the active matter. Dots—simulation results, lines—fits, according to the theoretical prediction (16). Here $f_c^{*} = f_c l_a/C_a$ is the reduced critical force and $\phi _a =\pi n l_a^2$ is the effective “packing fraction”. The parameters are $C_a=1$, $l_a=1.3$, $C_r=2$, $l_r=1.1$ and $D=0$. In simulations we use system sizes from $N=50$ to $N=2000$ and varying *b* from $b=0.0001$ to $b=100$. Note the qualitative agreement between the theory and simulations. (**b**) Internal structure of a swirlon. It is comprised of particles moving clockwise and counterclockwise.

The three phases of active matter—gaseous, liquid and swirlonic, obtained in simulations are demonstrated in Fig. 4a. If we assume that $\alpha _1$ is considerably larger than $\alpha$, we conclude that the observed phase diagram is in a qualitative agreement with the theoretical prediction (16).

### Conditions for collapsed state

Here we call a state with a single structure formed as a “collapsed” state. This may happen if a considerable amount of particles does not experience an action of a local fluctuating forces, that cause the emergence of local swirlons, but rather experience a “global” force, directed towards the center of the occupied area. In other words, it is expected that collapsed structures would be formed, provided that number of “boundary” particles, located in a layer with the distance of $l_a$ from the boundary is comparable to the number of “internal” particles. This condition may be written as follows,

17 $$\begin{aligned} \pi R^2 n - \pi (R-l_a)^2n \approx \pi (R-l_a)^2 n. \end{aligned}$$

If we neglect $l_a^2$ as compared to $Rl_a$ we can write approximate condition for the formation of a single structure:

18 $$\begin{aligned} l_a \approx \frac{1}{4} R . \end{aligned}$$

As one can see from the above equation, the collapsed state emerges in small systems, with a long range attraction. This tendency has been confirmed in our simulations.

## Swirlonic phase

### Swirlons under an external force

The active particles forming swirlons are orbiting the common center of mass in different directions: approximately half of them move clockwise and half—counterclockwise, which is illustrated in Fig. 4b; still, they move coherently. Namely, in our simulations of active matter we observe that swirlons (in the swirlonic phase) move under an action of external force, as material particles in viscous medium. That is, their velocity linearly increases with the applied force. The mobility of a swirlon depends on its mass, that is, on the number of active particles comprising the swirlon. The larger the mass of a swirlon the smaller the mobility.

Below we explain this astonishing property of swirlons. Let a constant external force ${\varvec{g}}$ is directed along the *x*-axis. Let a swirlon be in a steady motion with a constant velocity $v_0$, also along the *x*- axis. Consider a motions of an active particle inside the swirlon. Let ${\varvec{F}}$ be the resulting total force that acts on the particle from the other particles of the swirlon. It supports the circular motion; the force and velocity components may be written as:

19 $$\begin{aligned} F_x & = F\cos \omega t ; \qquad F_y= F \sin \omega t \end{aligned}$$

20 $$\begin{aligned} v_x & = v_1 \cos \omega t +v_0 ; \qquad v_y= v_1 \sin \omega t , \end{aligned}$$

where $\omega$ is the angular frequency and $v_1$ is the linear velocity of the circular motion . The *x*-component of the equation of motion then reads,

21 $$\begin{aligned} \dot{v}_x = F_x +(b-v^2)v_x +g . \end{aligned}$$

Now we take into account that

$$\begin{aligned} v^2 & = v_x^2 +v_y^2 =v_1^2 +v_0^2 +2 v_1 v_0 \cos \omega \\ v^2v_x & = v_1^3 \cos \omega t +v_1^2 v_0 +v_1v_0^2 \cos \omega +v_0^3 + 2 v_1^2 v_0 \cos \omega t +2 v_1 v_0^2 \cos \omega t \end{aligned}$$

and average over time *T* which is large, as compared with the rotational period $T_{swir}=2\pi /\omega$. Introducing the notation,

$$\begin{aligned} \overline{a} = \frac{1}{T} \int _0^T a(t) dt, \end{aligned}$$

we obtain,

22 $$\begin{aligned} \overline{v^2} & = v_1^2 +v_0^2 \nonumber \\ \overline{v^2 v_x} & = 2v_1^2v_0 +v_0^3 \nonumber \\ \overline{\dot{v}_x} & = \overline{f_x } =0 \nonumber \\ \overline{v_x} & = v_0. \end{aligned}$$

Averaging the equation of motion (21) we find,

23 $$\begin{aligned} \overline{\dot{v}_x} = \overline{f_x} + \overline{(b-v^2) v_x} + \overline{g} , \end{aligned}$$

or, with Eqs. (22) and (23),

24 $$\begin{aligned} bv_0 -(2v_1^2v_0 +v_0^3)+ g =0, \end{aligned}$$

which is a cubic equation for $v_0$. Almost in all cases the constant velocity $v_0$ is much smaller than the orbiting velocity $v_1$ (that is, $v_0 \ll v_1$), which implies the approximation, $v_1^2 \simeq v^2 \simeq \left\langle v^2 \right\rangle$, where $\left\langle v^2 \right\rangle$ is the mean square velocity of particles in the swirlon. Then the solution of Eq. (24) may be written as

25 $$\begin{aligned} v_0 =\frac{g}{2\left\langle v^2 \right\rangle -b}. \end{aligned}$$

**Figure 5:**
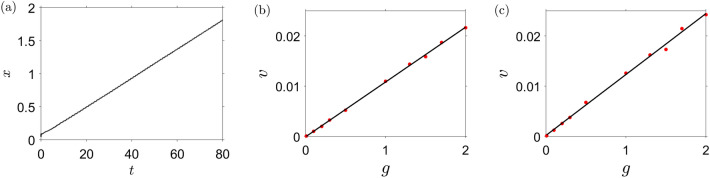
Uniform motion of swirlons under an external load: (**a**) The linear dependence on time of the displacement of the center of mass of a swirlon under an action of the external force. The measured velocity is $v \simeq 0.0216$ and the theoretical value is $v \simeq 0.0211$. (**b**,**c**) The linear dependence of the velocity of the swirlon *v* on the magnitude of the applied force *g*. Dots—simulation results, curves—theoretical prediction, Eq. (25); $\left\langle v^2 \right\rangle$ is taken from the simulations. The parameters are $C_a=5$, $l_a=15$, $C_r=1$, $l_r=0.01$, $b=2$, $D=0.1$ and $N=200$ for (**a**,**b**) and $C_a=5$, $l_a=15$, $C_r=1$, $l_r=2$, $b=1$, $D=0.1$ and $N=200$ for (**c**).

Note that the orbiting velocity $v_1$ may strongly differ from the “self-velocity” $b^{1/2}$. Indeed, when a swirlon is formed, the initial potential energy of particles may transform into the kinetic energy associated with the orbiting motion around the center of mass of the swirlon. It is not easy, however, to obtain the value of $\left\langle v^2 \right\rangle$, due to the lack of conservation laws for energy, momentum and angular momentum for active particles. In Fig. 5 the simulation results are compared to the theoretical prediction, Eq. (25). The theoretical results are in a very good agreement with the numerical data.

### Coalescence of swirlons

**Figure 6:**
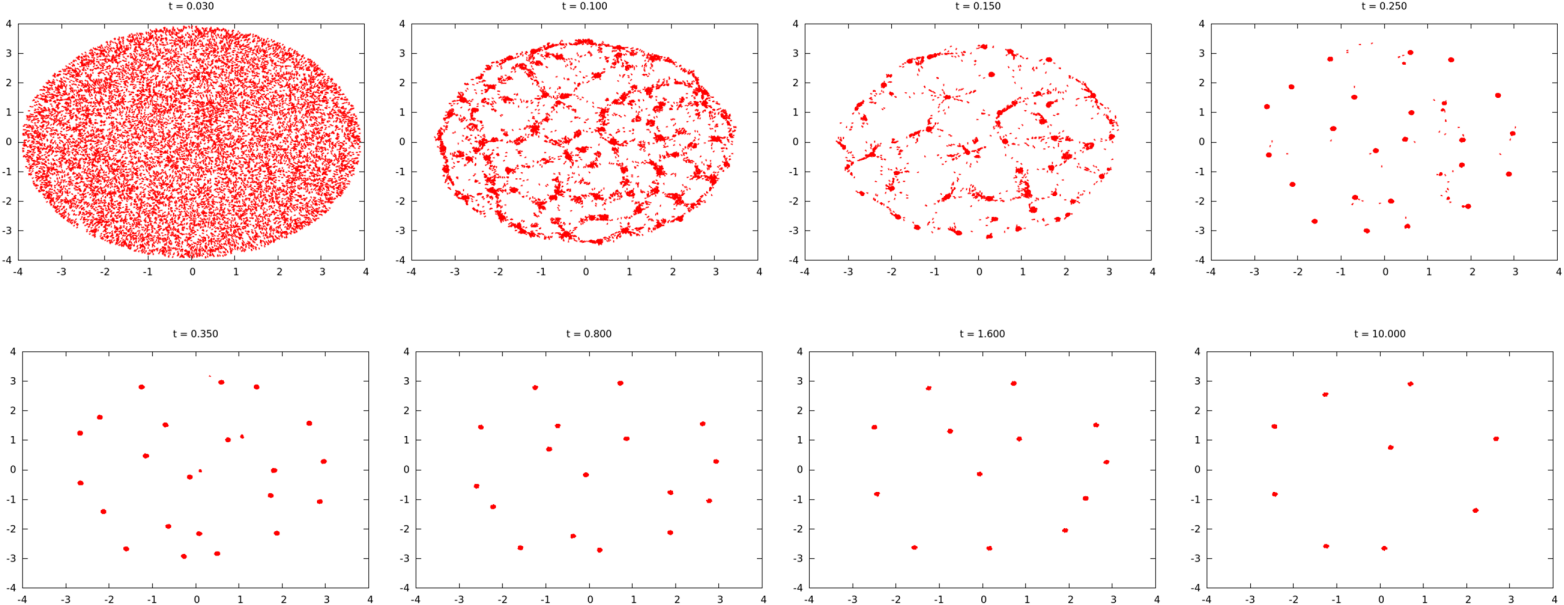
Evolution of the swirlonic state. The initial number of active particles is $N=16{,}000$, which were uniformly distributed in a circle of radius $R_0=4$ The parameters are $C_a=10$, $l_a=0.2$, $C_r=1$, $l_r=0.1$, $b=20$, $D=0$. The coalescence is fast for swirlon of small mass and becomes significantly slower when their mass increases, which is detailed in Fig. 7.

In Fig. 6 we demonstrate the main stages of the swirlon emergence and evolution. One can observe the coalescent dynamics in the swirlonic phase. Initially the aggregation is fast, since the swirlons attract each other and have large mobility. In a course of time the average mass of swirlons increases and the mobility drops down (see the discussion below). This results in the slowing down of the coalescence. Eventually a small amount of very massive swirlons remain that have vanishing mobility and the aggregation practically ceases.

Consider some particular stage of the swirlon evolution. Let $N_s$ swirlons emerge in a system with the area *S* and the initial number density of active particles be *n*. Then the average “mass” of a swirlon (that is, the number of active particles forming a swirlon) reads, $m_s=nS/N_s$ and the number density of the swirlons is $n_s= N_s/S =n/m_s$. The average distance between the swirlons may be estimated as $\bar{l} \sim n_s^{-1/2}$. The coalescence of these objects occurs as a process that symbolically may be written as $A+A \rightarrow A$, where *A* denotes a single swirlon. We estimate the rate of this process. Let two swirlons were initially at the average distance $r(0)=\bar{l}$ and after time *T* coalesce, $r(T) \approx 0$. Their relative motion obeys the equation,

26 $$\begin{aligned} \frac{dr}{dt} = - \mu \frac{C_a m_s^2}{l_a} e^{-r/l_a}. \end{aligned}$$

where $\mu$ accounts for the mobility, which we approximate as size-independent and the factor $m_s^2$ accounts that in each swirlon there are $m_s$ monomers, each attracting with the force $(C_a/l_a)e^{-r/l_a}$. Solving Eq. (26) with the above boundary conditions we find:

27 $$\begin{aligned} e^{\bar{l} /l_a}= 1+\frac{\mu C_a m_s^2 }{l_a^2} T. \end{aligned}$$

Since $e^{\bar{l} /l_a} \gg 1$ we conclude that

$$\begin{aligned} T^{-1} = \frac{\mu C_a m_s^2}{l_a^2} e^{-\bar{l} /l_a}. \end{aligned}$$

The quantity $T^{-1}$ may be treated as the reaction rate between two swirlons. Therefore the equation for the reaction kinetics $A+A \rightarrow A$ reads

$$\begin{aligned} \frac{d n_s}{dt} & = -\frac{1}{2} T^{-1} n_s^2 = -\frac{1}{2} \mu \frac{C_a m_s^2}{l_a^2}n_s^2 e^{-\bar{l} /l_a} = -\frac{1}{2} \frac{\mu C_a n^2}{l_a^2} e^{-1/(n_sl_a^2)^{1/2}}, \end{aligned}$$

where we use the expressions $m_s=n/n_s$ and $\bar{l}=1/n_s^{1/2}$. Solving the above equation we obtain for large *t* and small $n_sl_a^2 \rightarrow 0$,

$$\begin{aligned} x^{3/2}e^{1/\sqrt{x}} = \frac{1}{4} \mu C_a n^2 t, \end{aligned}$$

where $x=n_sl_a^2$ or, asymptotically, for $t \rightarrow \infty$,

28 $$\begin{aligned} n_s \sim \frac{1}{\left( \ln t \right) ^2}. \end{aligned}$$

**Figure 7:**
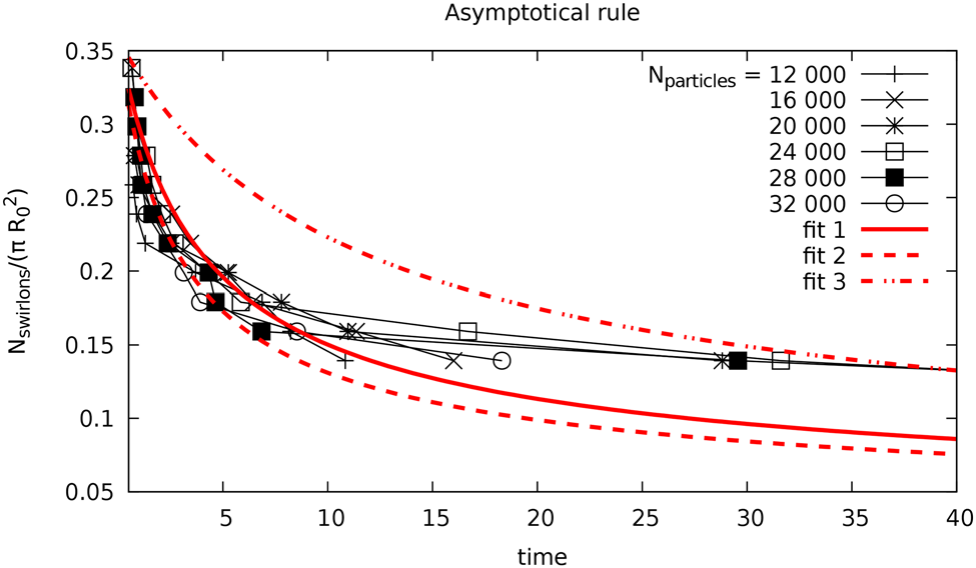
Time dependence of the swirlon number density. It decreases owing to the permanent coalescence of the swirlons. In a course of time this process becomes extremely, slow, as it follows from Eq. (28). Dots—simulation data for the different system size, lines—fits, corresponding to the theoretical expression (28): fit 1: $0.358 / (\log _{10}(2.5 t+10))^2$, fit 2: $0.358 / (\log _{10}(3.5 t+10))^2$, fit 3: $0.358 / (\log _{10}(0.85 t+10))^2$. The numerical data resides between the according analytical fits. The parameters are $C_a=10$, $l_a=0.2$, $C_r=1$, $l_r=0.1$, $b=20$, $D=0$ and $R_0=4$.

Eq. (28) predicts an extremely slow decay of the swirlons concentration, which agrees qualitatively with the simulation results, as it is illustrated in Fig. 7.

It is interesting to compare the coalescence of swirlons with the aggregation of vertexes in two-dimensional turbulence, although the background physics of these two phenomena is rather different. The vertex aggregation results in a power-law decay of a number of vertexes, $n_v \sim t^{-\xi }$ with $\xi \approx 0.70 - 0.75$^57^, which is much faster than the inverse square logarithm of time in Eq. (28). This is explained by the long-range interaction of the vertexes, while the swirlon interactions decay exponentially with distance.

## Conclusion

We reveal a new state of active matter, which we call a swirlonic state, where all active particles belong to swirlons, comprised of particles orbiting around common center of mass. Although such milling structures have been reported in many studies, the state of matter constituted by quasi-particles—swirlons was not investigated. The dimension of swirlons is almost independent on the number of particles inside it and determined by the intensity of the self-driven and inter-particle forces. These quasi-particles demonstrate an astonishing property—under an applied external force they move with a constant velocity, proportional to the force, just as the objects in viscous medium. We observe that the mobility of a swirlon is inversely proportional to the square average velocity of particles orbiting the swirlon’s center of mass. Swirlons attract to each other and coalesce upon a collision, forming a joint swirlon. Therefore evolution of a swirlonic state of matter corresponds to continuous aggregation of particles when fewer and fewer swirlons of larger and larger mass are formed. This process being fast in the beginning, becomes extremely slow afterwards, leading to the formation of a rarified state of massive immobile swirlons; the evolution at this stage is practically frozen.

We also observe, that depending on the parameters of the inter-particle potential and characteristics of the self-driving force of active particles, a few other states may be formed. These correspond to the gaseous, liquid and solid states of matter, as it is known for molecular systems. While in the gaseous state the active matter occupies all the available space, in the liquid state only a part of the space is occupied. Moreover, in the liquid state the active matter possesses a surface tension. Surprisingly, we did not observe the coexistence between liquid and gaseous phase, as it is common for molecular systems. We explain this feature of the active matter by the lack of fast particles, which can escape from the liquid phase and form the gaseous phase. While the abundance of fast particles is secured in molecular systems by the Maxwellian velocity distribution, the velocity distribution for fast particles in active matter has much steeper decay with increasing velocity.

For high density of active particles with a small self-driving force and relatively strong repulsive interactions, a solid state is formed. It is characterized by a complete suppression of the long-range motion (diffusion) of the particles. In the solid state the active particles perform a very limited motion around a center of a potential well formed by their nearest neighbors. Finally, for the case of a low self-driving force and very long-range attractive interactions the active matter forms a collapsed state. This is a dense state where we have observed quite irregular motion of the particles.

We performed both numerical and theoretical analysis of the system. To make the set of ODEs, that describe the particles motion, less stiff, we use a modified Morse potential, which has the gaussian form of its repulsive part. This allows to simulate a few tens of thousands of equations, which suffices to investigate different macroscopic states of the system. The qualitative and quantitative predictions of our theory are in a good agreement with the simulation results.

## Appendix

### Fokker–Plank equation and the velocity distribution

Application of the standard techniques^55, 56^ yields the following Fokker-Plank equation for the distribution function $\Phi ({\varvec{r}}, {\varvec{v}})$ for the coordinates and densities of active particles:

29 $$\begin{aligned} \frac{\partial \Phi }{\partial t} + {\varvec{v}} \cdot \frac{\partial \Phi }{\partial {\varvec{r}}} + {\varvec{g}} \cdot \frac{\partial \Phi }{\partial {\varvec{v}}} =-\frac{\partial }{\partial {\varvec{v}}} \cdot ( b-v^2) {\varvec{v}} \Phi + \frac{D}{2} \frac{\partial ^2 \Phi }{\partial {\varvec{v}} \partial {\varvec{v}}}, \end{aligned}$$

where as previously ${\varvec{g}}$ is the external force. We consider the stationary distribution for a space uniform system in the absence of external forces. In this case the l.h.s of Eq. (29) vanishes, yielding,

30 $$\begin{aligned} {\mathrm{div}}_v \left[ (b-v^2) {\varvec{v}} \, \Phi \right] = \frac{D}{2}\Delta _v \Phi . \end{aligned}$$

For uniform and isotropic system the distribution function $\Phi$ depends only on velocity module, that is, $\Phi = \Phi (v)$. Changing to the polar coordinates for the velocity, ${\varvec{v}} =(v, \phi )$, we obtain, using the independence of $\Phi$ on the angular variable $\phi$:

31 $$\begin{aligned} \frac{1}{v} \frac{\partial }{\partial v} \left[ v (b-v^2) v \Phi \right] = \frac{D}{2} \frac{1}{v} \frac{\partial }{\partial v} v \frac{\partial \Phi }{\partial v}. \end{aligned}$$

Integration this equation and using the boundary condition at $v=0$, we finally obtain:

32 $$\begin{aligned} \Phi \sim e^{bv^2/D - v^4/2D}, \end{aligned}$$

which for $v \rightarrow \infty$ tends to $\Phi \sim e^{-v^4/2D}$.

### Derivation of the force distribution

Following the derivation of the Holtsmark distribution given for the gravitational force $f(r) \sim 1/r^2$ (see e.g.^59^), we write for the distribution of the local force ${\varvec{F}}$:

33 $$\begin{aligned} P({\varvec{F}}) = \int \frac{d {\varvec{r}}_1}{\Omega } \ldots \int \frac{d {\varvec{r}}_N}{\Omega }\delta \left( \sum _{i=1}^N {\varvec{f}}_i -{\varvec{F}} \right) , \end{aligned}$$

where $\Omega$ is the system volume and *N* is the number of particles. Now we take a Fourier transform of $P({\varvec{F}})$ to obtain:

$$\begin{aligned} P({\varvec{k}})= & \int d {\varvec{F}} e^{i {\varvec{k}} \cdot {\varvec{F}}} P({\varvec{F}}) = \int \frac{d {\varvec{r}}_1}{\Omega } \ldots \int \frac{d {\varvec{r}}_N}{\Omega } e^{i {\varvec{k}} \cdot {\varvec{F}}} \delta \left( \sum _{i=1}^N {\varvec{f}}_i -{\varvec{F}} \right) = \left[ \int \frac{d {\varvec{r}}}{\Omega } e^{i {\varvec{k}} \cdot {\varvec{f}}} \right] ^N. \end{aligned}$$

In the limit $\Omega \rightarrow \infty$, $N \rightarrow \infty$, $n=N/\Omega =\mathrm{const.}$ the last result takes the form^59^,

34 $$\begin{aligned} P({\varvec{k}}) =\exp \left[ -n \int d {\varvec{r}} \left( 1- e^{i {\varvec{k}} \cdot {\varvec{f}}} \right) \right] =e^{-n \Phi ({\varvec{k}})}, \end{aligned}$$

which defines $\Phi ({\varvec{k}})$. Hence we obtain for the distribution function,

35 $$\begin{aligned} P({\varvec{F}}) = \left( \frac{1}{2 \pi } \right) ^2 \int d {\varvec{k}} e^{-i{\varvec{k}} \cdot {\varvec{F}} - n \Phi ({\varvec{k}})} . \end{aligned}$$

To compute $\Phi ({\varvec{k}})$ we write the force as ${\varvec{f}}({\varvec{r}}) = ({\varvec{r}}/r) f_1(r)$ with $f_1(r)=(C_a/l_a) re^{-r/l_a}$. Then one obtains for the two-dimensional system:

36 $$\begin{aligned} \Phi ({\varvec{k}})= 2 \int _0^{\pi }d \phi \int _0^{\infty } r dr \left( 1-e^{ikrf_1(r) \cos \phi } \right) . \end{aligned}$$

Integration over $\phi$ yields,

$$\begin{aligned} \int _0^{\pi }d \phi \left( 1-e^{ikrf_1(r) \cos \phi } \right) & = \pi \left[ 1- J_0\left( krf_1(r)\right) \right] = \pi \left[ 1- J_0 \left( \frac{kC_a}{l_a}e^{-r/l_a} \right) \right] , \end{aligned}$$

where $J_0(x)$ is the zero-order Bessel function. Integration over *r* in (36) may not be performed analytically, hence we apply approximations. To analyze capturing of particles by the fluctuating force we are interested in the force distribution for large forces, that exceed the characteristic force of the order of $\sim C_a/l_a$ . In the Fourier space this would correspond to values of $k \sim 1/F$ smaller than $\sim (C_a/l_a)^{-1}$. In other words, we are interested in the values of *k* satisfying the relation, $kC_a/l_a <1$. This motivate us to apply the approximation, $J_0(x) \simeq 1- \frac{1}{4} x^2$ with $x=(kC_a/l_a)e^{-r/l_a}$, which yields,

37 $$\begin{aligned} \Phi ({\varvec{k}})\approx & 2 \pi \int _0^{\infty } r dr \left( 1-1+\frac{1}{4} k^2 \frac{C_a^2}{l_a^2} e^{-2 r/l_a}\right) = \frac{\pi }{2} \frac{C_a^2}{l_a^2} k^2 \left( \frac{l_a}{2} \right) ^2 \int _0^{\infty } z e^{-z} dz = \frac{\pi }{8} k^2 C_a^2 . \end{aligned}$$

Using Eq. (37) we find the distribution of the fluctuating forces:

$$\begin{aligned} P({\varvec{F}})= & \frac{2}{(2\pi )^2} \int _0^{\infty } k dk \int _0^{\pi } e^{-ikF \cos \phi -nak^2} = \frac{2}{4 \pi ^2}\int _0^{\infty } k dk e^{-nak^2} \int _0^{\pi } e^{-ikF \cos \phi }\\= & \frac{1}{2 \pi }\int _0^{\infty } k dk e^{-nak^2} J_0(kF) = \frac{1}{2 \pi } \frac{e^{-F^2/4an}}{2 an} , \end{aligned}$$

where $a= \pi C_a^2/8$. It may be easily checked that $P({\varvec{F}})$ is normalized and that $\left\langle {\varvec{F}} \right\rangle = \int d{\varvec{F}} P({\varvec{F}}) {\varvec{F}} =0$.
